# Trastuzumab deruxtecan in an elderly patient with HR+/HER2-low breast cancer complicated by pleural effusion: a case report and literature review

**DOI:** 10.3389/fonc.2025.1728983

**Published:** 2025-12-18

**Authors:** Shiyu Huang, Saikam Law, Maojian Chen, Hongna Lai

**Affiliations:** 1 Guangdong Provincial Key Laboratory of Malignant Tumor Epigenetics and Gene Regulation, Guangdong-Hong Kong Joint Laboratory for Ribonucleic Acid (RNA) Medicine, Breast Tumor Center, Sun Yat-Sen Memorial Hospital, Sun Yat-Sen University, Guangzhou, China; 2 Department of Gastrointestinal Surgery, Sun Yat-sen Memorial Hospital, Sun Yat-sen University, Guangzhou, China

**Keywords:** trastuzumab deruxtecan, HR+/HER2-low, breast cancer, elderly, pleural effusion

## Abstract

Trastuzumab deruxtecan (T-DXd or DS-8201) is a novel antibody–drug conjugate (ADC) targeting HER2 that has shown significant efficacy in advanced breast cancer. However, data on its use in elderly patients with hormone receptor–positive (HR+)/HER2-low disease complicated by malignant pleural effusion are scarce. We report the case of a 75-year-old woman with HR+/HER2-low (HER2 2+/FISH– at diagnosis, HER2–0 at recurrence) advanced breast cancer who developed multiple lymph node metastases and a large pleural effusion after progression on endocrine therapy plus a CDK4/6 inhibitor. She received trastuzumab deruxtecan as salvage therapy, initially at a reduced dose of 4.4 mg/kg because of frailty, followed by escalation to the standard 5.4 mg/kg dose after good tolerability. Several cycles were administered at extended intervals due to financial constraints. T-DXd therapy resulted in near-complete resolution of the pleural effusion, marked shrinkage of nodal disease, improvement of upper limb edema and respiratory symptoms, and sustained declines in serum tumor markers. Progression-free survival on T-DXd reached 16 months, while overall survival from T-DXd initiation was 18 months. Treatment-related adverse events were limited to mild myelosuppression and gastrointestinal discomfort, without interstitial lung disease. This case suggests that dose-adjusted T-DXd can achieve durable disease control with acceptable safety in a frail elderly patient with HR+/HER2-low breast cancer and pleural effusion, adding to the growing real-world evidence supporting the use of HER2-directed ADCs beyond traditional HER2-positive populations.

## Introduction

Breast cancer is the most common malignancy and the second leading cause of cancer-related death among women worldwide, posing a major threat to women’s health and longevity (1). To improve disease evaluation and guide clinical decision-making, breast cancer is classified into three molecular subtypes based on ER, PR, and HER2 immunohistochemistry: HR+/HER2-, HER2+, and triple-negative breast cancer. Among them, the HR+/HER2- subtype accounts for approximately 50–60% of all breast cancer cases in China (2). For patients with metastatic HR+/HER2- breast cancer, a combination of CDK4/6 inhibitors and endocrine therapy is recommended as the first-line treatment. However, optimal therapeutic strategies following CDK4/6 inhibitor resistance remain unclear.

Historically, this subtype was considered unresponsive to HER2-targeted therapy. However, with the recognition that some patients exhibit low levels of HER2 expression, novel anti-HER2 antibody–drug conjugates (ADCs) have demonstrated potential benefits in this subgroup. This led to the establishment of the term “HER2-low,” which refers to tumors with HER2 immunohistochemistry (IHC) 1+ or IHC 2+ and ISH-negative results, while cases with IHC 0 are classified as HER2-negative (3, 4). The pivotal DESTINY-Breast04 trial first demonstrated that the HER2-targeted ADC trastuzumab deruxtecan (T-DXd) significantly improved progression-free survival (PFS) and overall survival (OS) in patients with HER2-low metastatic breast cancer (5). Subsequent studies, such as DAISY and DS-8201-A-J101, further confirmed these findings.

This case provides real-world evidence supporting the efficacy and tolerability of trastuzumab deruxtecan (DS-8201) as a salvage therapy in an elderly patient with HR+/HER2-low advanced breast cancer, demonstrating favorable tumor response and manageable adverse effects.

## Case presentation

### Basic information and initial diagnosis

A 75-year-old woman with a history of uterine leiomyoma treated by total hysterectomy in 1998, with no other significant medical history, presented to Sun Yat-sen Memorial Hospital in January 2022 with a mass in the left breast that had been noted six months earlier. Physical examination revealed ulcerated and bleeding skin in the outer quadrant of the left breast, discharging bloody, purulent, pale-yellow fluid, with surrounding skin thickening and induration.

**Figure 1:**
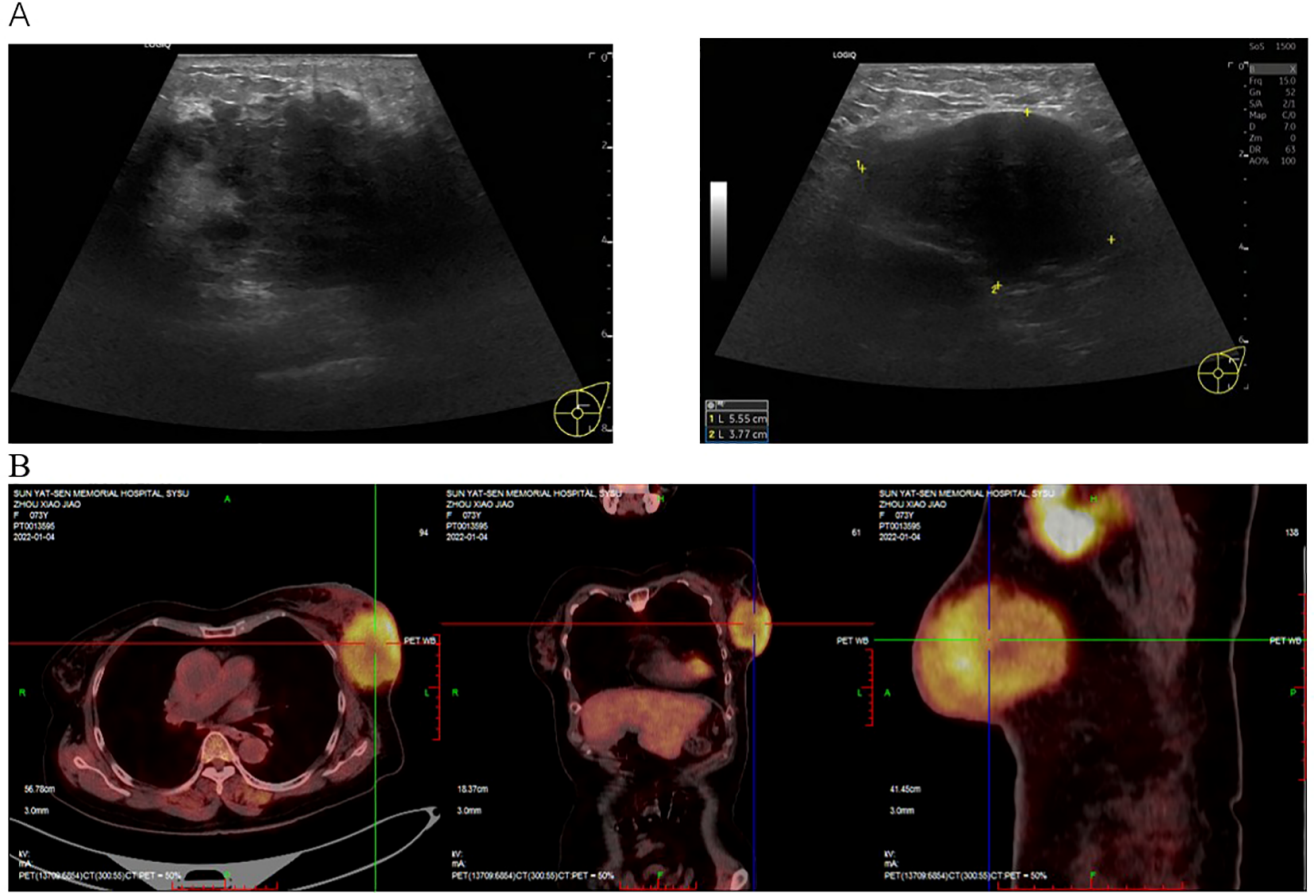
Imaging findings at the patient’s initial visit. **(A)** Breast ultrasound reveals thickened skin over the left breast, subcutaneous tissue edema, and a large mass (approximately 5+ × 3.9 × 5+ cm in size). Multiple lymph node echogenic foci, the largest measuring 5.6 × 3.8 cm, suggestive of metastasis. **(B)** PET-CT reveals a mass in the outer quadrant of the left breast, suspected to be breast cancer, with multiple lymph node metastases.

Breast ultrasound and PET-CT were performed. Ultrasound revealed diffuse skin thickening of the left breast and a large hypoechoic mass (approximately 5.0 × 3.9 × 5.0 cm) extending from the subcutaneous tissue to the deep glandular layer. Multiple enlarged lymph nodes with mixed echogenicity were detected in the left axillary, mid-axillary, and superior axillary regions, the largest measuring 5.6 × 3.8 cm. PET-CT demonstrated a metabolically active mass in the outer quadrant of the left breast consistent with breast cancer, with multiple hypermetabolic lymph nodes in the left axilla and intercostal muscles of the chest wall. Several metabolically active lymph nodes were also noted in the right axilla, raising suspicion of contralateral metastasis (**Figure 1**).

On January 10, 2022, a needle biopsy of the left breast mass was then performed. Pathology confirmed invasive carcinoma of the left breast. Immunohistochemical staining results showed: Estrogen Receptor (ER) 95%, Progesterone Receptor (PR) 85%, Her-2 (2+), Ki67 proliferation index 60%, FISH (–).

**Figure 2:**
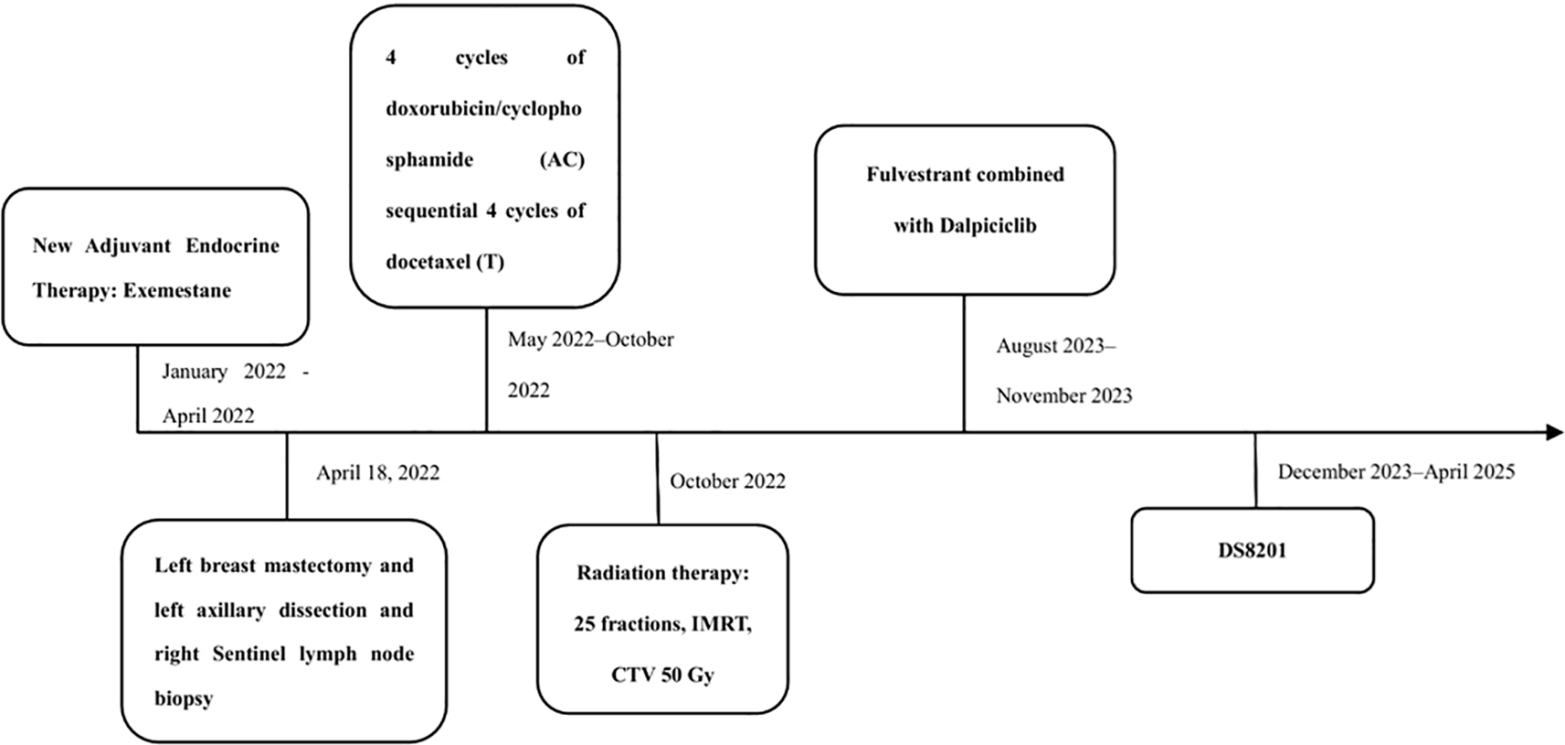
Patient’s treatment timeline.

Comprehensive evaluation confirmed Luminal B1 left breast cancer, stage cT4N3M0. Anticancer treatment commenced in January 2022, with the patient having received multiple lines of therapy. Treatment details are summarized in **Figure 2**.

### Neoadjuvant endocrine therapy

After detailed discussion, the patient understood her condition and treatment options but declined chemotherapy. On January 12, 2022, she initiated neoadjuvant endocrine therapy with exemestane (25 mg orally once daily). By April 2022, the tumor and the ulcerated surface had markedly regressed, achieving a partial response (PR). Breast MRI demonstrated a 6.4 × 4.3 × 6.0 cm mass in the outer quadrant of the left breast involving the overlying skin, consistent with breast cancer, along with multiple metastatic lymph nodes in the left axilla and between the pectoralis major and minor muscle spaces. Several right axillary lymph nodes were also enlarged, raising suspicion of metastasis.

### Surgery

On April 18, 2022, the patient underwent a modified radical mastectomy of the left breast with left axillary lymph node dissection (ALND) and right sentinel lymph node biopsy (SLNB). Postoperative pathology revealed grade III invasive carcinoma of unspecified type infiltrating the dermis, with metastases in 9 of 13 left axillary lymph nodes. Immunohistochemistry of the left breast specimen showed ER 90% positive, PR 10% positive, HER2 (1+), and a Ki-67 proliferation index of 25%. The right sentinel lymph node was negative for metastasis. The final pathological stage was ypT4N2M0, corresponding to Stage IIIB.

### Postoperative adjuvant therapy

Because of enlarged contralateral axillary lymph nodes, metastasis could not be excluded. The patient was advised to continue adjuvant therapy following surgery. On May 12, 2022, she initiated first-line adjuvant chemotherapy consisting of four cycles of AC (liposomal doxorubicin 50 mg + cyclophosphamide 940 mg, every 3 weeks), followed by four cycles of T (albumin-bound paclitaxel 400 mg, every 3 weeks), in combination with 25 sessions of radiotherapy.

During surveillance, an ultrasound on July 14, 2022, revealed multiple lymph node masses in the left axillary, supra-axillary, and bilateral supraclavicular regions, measuring approximately 1.1 × 0.6 cm, 0.7 × 0.5 cm (right supraclavicular), and 0.8 × 0.6 cm (left supraclavicular). After completion of eight chemotherapy cycles, follow-up imaging showed persistent lymph node masses in the same regions, with sizes of about 0.9 × 0.6 cm (left axillary), 0.9 × 0.4 cm (right supraclavicular), and 0.7 × 0.4 cm (left supraclavicular), corresponding to stable disease (SD). Subsequently, the patient declined further endocrine therapy and discontinued regular follow-up visits for personal reasons.

### Recurrence and metastasis phase

In August 2023, left axillary lymphadenopathy and ipsilateral upper limb edema were observed. Ultrasound and CT imaging revealed multiple enlarged lymph nodes in both axillae. A biopsy of the left axillary lymph node confirmed invasive carcinoma. Immunohistochemistry demonstrated ER 90% positive, PR 85% positive, HER2 (0), and Ki-67 proliferation index 45%. The diagnosis was established as stage IV recurrent invasive breast carcinoma, Luminal B1 subtype, with a disease-free survival (DFS) of 16 months. Chest CT additionally showed a moderate left pleural effusion, mild compression atelectasis in the left lower lobe, and radiation-induced pneumonia in the left anterior pleura, accompanied by slight atelectasis in the lingular segment of the left upper lobe.

### Initial rescue treatment

**Figure 3:**
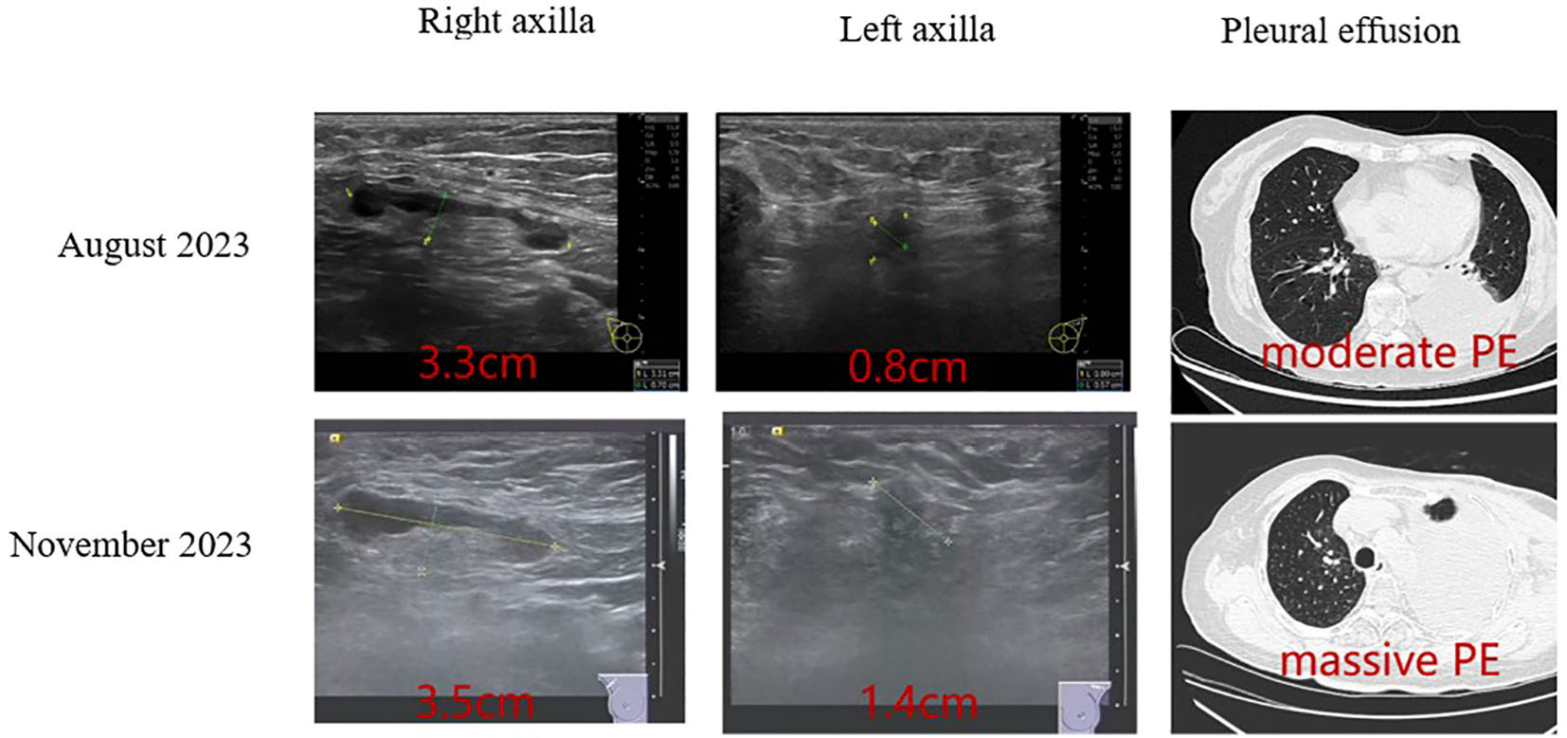
Comparison of treatment outcomes before and after combination therapy with fulvestrant and dalpiciclib. Before: August 2023, After: November 2023.

In August 2023, the patient began first-line systemic therapy for ER+/HER2-low advanced breast cancer, consisting of the CDK4/6 inhibitor dalpiciclib (125 mg orally once daily) combined with the aromatase inhibitor fulvestrant (500 mg intramuscularly every 4 weeks). After three treatment cycles, the patient exhibited persistent lymphadenopathy, increased pleural effusion volume (**Figure 3**), and continuously rising serum tumor markers (CEA, CA19-9, and CA15-3), indicating disease progression. The progression-free survival (PFS) was 3 months.

### Subsequent rescue treatment

**Figure 4:**
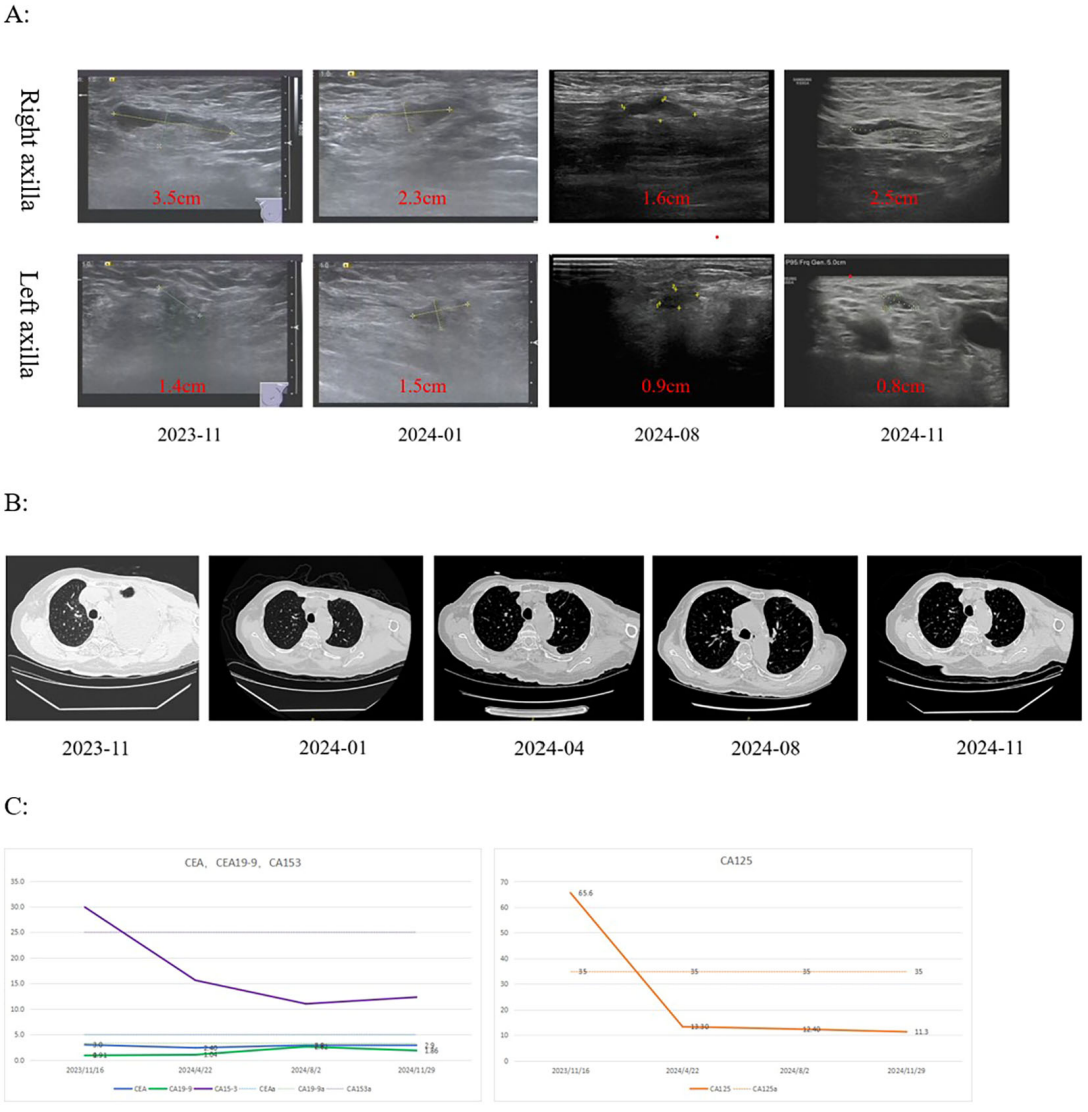
Trends in imaging and biomarker changes during treatment with antibody-drug conjugates. **(A)** axillary lymph node ultrasound scan. **(B)** Chest CT demonstrates changes in the amount of pleural effusion. **(C)** trends in different biomarker.

On December 5, 2023, the patient commenced treatment with the antibody–drug conjugate trastuzumab deruxtecan (T-DXd). In our elderly patient, we elected to begin at a slightly reduced dose (4.4 mg/kg) out of an abundance of caution, given her age (82 years) and frail condition. This initial dose corresponds to the first dose-reduction level defined in dosing guidelines. After confirming good tolerance (only Grade 1 hematologic and gastrointestinal adverse events according to CTCAE v5.0), the dose was escalated to the standard 5.4 mg/kg from the third cycle onward. Importantly, no efficacy compromise was observed – she achieved a notable tumor response – and this careful approach may have averted early high-grade toxicity. Because of financial constraints, several treatment cycles were administered at 28-day intervals rather than the standard 21-day schedule. Strictly speaking, guidelines do not endorse prolonged intervals – even patients over 65 are generally treated on the same Q3W. However, real-world oncology practice sometimes adapts treatment schedules for patient-centered reasons when efficacy is maintained. In our revision, we note that this patient’s disease remained under control. Following three courses of treatment, the pleural effusion had largely resolved and did not manifest again thereafter. By August 2024, the primary lesion had decreased in size by approximately 50%, achieving a partial response (PR). Upper limb swelling resolved, lung function improved, and serum tumor markers (CEA, CA19-9, and CA15-3) steadily declined during therapy (**Figure 4**). During the therapeutic regimen, treatment-related adverse events included mild (Grade 1–2) myelosuppression and gastrointestinal discomfort, without evidence of interstitial lung disease (ILD). The final administration of T-DXd was given on April 29, 2025.

However, from May 2025 onward, the patient’s condition deteriorated rapidly, presenting with impaired consciousness and aphasia. Cranial MRI performed at a local hospital suggested possible brain metastases. The patient passed away on June 29, 2025. The total duration of T-DXd therapy was 16 months, with an overall survival of 18 months from treatment initiation.

## Discussion

This case illustrates that trastuzumab deruxtecan (T-DXd) can provide meaningful clinical benefit in an elderly, frail patient with hormone receptor–positive (HR+)/HER2-low breast cancer and malignant pleural effusion, even though the recurrent biopsy was HER2-0. HER2-low disease, defined as HER2 immunohistochemistry (IHC) 1+ or 2+ with negative *in situ* hybridization, has recently been recognized as a therapeutically relevant subset, and expert consensus now recommends that such patients be considered for HER2-directed ADCs (6–9). DESTINY-Breast04 first showed that T-DXd significantly improved progression-free survival (PFS) and overall survival (OS) compared with chemotherapy in HER2-low metastatic breast cancer (5). DESTINY-Breast06 extended this benefit to endocrine-resistant HR+/HER2-low and HER2-ultralow disease, reporting a median PFS of about 13 months in the HER2-low cohort versus 8 months with physician’s-choice chemotherapy (10). Our patient’s 16-month PFS on T-DXd is in line with these pivotal data, despite substantial comorbidity, prior multimodality therapy and pleural effusion.

The apparent discrepancy between HER2-low expression in the primary tumor and HER2–0 in the recurrent lymph node highlights intratumoral heterogeneity and temporal evolution. T-DXd consists of a humanized anti-HER2 monoclonal antibody linked via a cleavable tetrapeptide linker to a highly potent topoisomerase I inhibitor payload, with a relatively high drug-to-antibody ratio (11). After binding to HER2 and internalization, the linker is cleaved and the membrane-permeable payload is released, allowing diffusion into adjacent tumor cells with very low or undetectable HER2 expression and generating a “bystander effect” (11). Together with sampling error on re-biopsy, this mechanism provides a biologically plausible explanation for the sustained response observed in a patient whose metastatic specimen was formally HER2-0.

T-DXd has a distinctive toxicity profile, with nausea, myelosuppression and alopecia being common, and interstitial lung disease (ILD) or pneumonitis recognized as the most serious adverse events (11, 12). A pooled analysis of nine T-DXd monotherapy trials reported an ILD incidence of approximately 12–15%, the majority being grade 1–2, but with a small proportion of grade ≥3 events and fatal cases (13). Current recommendations advocate systematic baseline chest imaging, regular symptom review, and prompt investigation of new respiratory complaints, using the Common Terminology Criteria for Adverse Events (CTCAE) v5.0 to guide grading and management (14). In our patient, advanced age, prior thoracic radiotherapy and the presence of pleural effusion all raised concern for ILD risk. We therefore chose a cautious dosing strategy with an initial reduction to 4.4 mg/kg, close clinical and radiologic monitoring, and subsequent escalation to the standard 5.4 mg/kg only after tolerability had been established. No ILD occurred during 13 cycles of therapy, underscoring the importance of proactive surveillance and early dose adjustment in high-risk patients.

Treatment decision-making for elderly breast cancer patients is inherently complex and requires careful consideration of comorbidities, drug toxicity, life expectancy, and patient preferences (15). As China’s aging population continues to grow, the number of elderly breast cancer patients will increase substantially (16, 17). Older and frail patients are markedly under-represented in pivotal trials, but accumulating real-world data suggest that T-DXd can be delivered safely to this population. Observational cohorts and registry studies, including elderly-focused series such as TREX-Old, indicate that efficacy in routine practice is broadly comparable to that seen in clinical trials, with median PFS often in the 10–15-month range and no major excess of severe toxicity when careful monitoring and timely dose modifications are implemented (18, 19). In line with these findings, our patient—aged 75 years with limited physiological reserve—derived a durable benefit from T-DXd with only low-grade hematologic and gastrointestinal adverse events. This supports the view that chronological age alone should not preclude T-DXd use when a geriatric assessment and shared decision-making process are in place.

The therapeutic landscape for endocrine-resistant HR+/HER2-negative metastatic breast cancer now includes several ADCs. Sacituzumab govitecan, a Trop-2–directed ADC, improved PFS (5.5 vs 4.0 months) and OS (14.4 vs 11.2 months) over single-agent chemotherapy in the phase III TROPiCS-02 trial of heavily pretreated HR+/HER2-negative disease, with a manageable toxicity profile dominated by neutropenia and diarrhea (20). Datopotamab deruxtecan, another Trop-2–directed ADC with a different payload, has also demonstrated significantly prolonged PFS compared with investigator’s-choice chemotherapy in the TROPION-Breast01 trial, albeit without a statistically significant OS advantage at the time of reporting (21). These agents provide important treatment options after endocrine therapy and chemotherapy, particularly for patients with truly HER2-null tumors or those who are ineligible for T-DXd.

Within this evolving ADC landscape, T-DXd is generally considered the preferred ADC for HER2-low disease after endocrine therapy failure, given the magnitude of benefit observed in DESTINY-Breast04 and DESTINY-Breast06 (5, 10). Sacituzumab govitecan and, where available, datopotamab deruxtecan are reasonable options after T-DXd or in patients who cannot receive T-DXd because of ILD risk or other contraindications (18–21). Our case underscores several practical lessons: historical pathology demonstrating HER2-low expression should be revisited when metastatic lesions appear HER2-0; dose and schedule of T-DXd can be individualized to balance efficacy and tolerability in older patients; and multidisciplinary teams play a crucial role in counselling, monitoring and sequencing ADCs.

This report is limited by its single-patient nature and the absence of comprehensive genomic profiling; however, it provides useful real-world insight into the individualized use of T-DXd in an elderly, frail patient with HR+/HER2-low breast cancer complicated by pleural effusion.

Overall, this case highlights the favorable clinical activity of T-DXd in managing HER2-low advanced breast cancer while underscoring the challenges of balancing efficacy and toxicity—particularly ILD—among elderly patients with multiple comorbidities.

## Conclusion

We present the case of an elderly female patient with HR+/HER2-low advanced breast cancer complicated by significant pleural effusion. After disease progression following multiple lines of therapy, treatment with trastuzumab deruxtecan (T-DXd, DS8201) demonstrated a meaningful antitumor response even in lesions with minimal HER2 expression. Following 13 cycles of therapy, a clinically meaningful reduction in tumor size was achieved. This case provides early real-world evidence supporting the clinical efficacy and tolerability of T-DXd in elderly patients with HER2-low breast cancer, underscoring its therapeutic potential in this challenging population.

## Data Availability

The raw data supporting the conclusions of this article will be made available by the authors, without undue reservation.
